# Height and risk of prostate cancer in the prostate, lung, colorectal, and ovarian cancer screening trial

**DOI:** 10.1038/sj.bjc.6605159

**Published:** 2009-06-30

**Authors:** J Ahn, S C Moore, D Albanes, W-Y Huang, M F Leitzmann, R B Hayes

**Affiliations:** 1 Division of Cancer Epidemiology and Genetics, National Cancer Institute, NIH, DHHS, Bethesda, MD 20852, USA; 2 Division of Epidemiology, New York University School of Medicine, New York, NY 10016, USA; 3 Department of Epidemiology and Preventive Medicine, University of Regensburg, Franz-Josef-Strauss-Allee 11, Regensburg D-93053, Germany

**Keywords:** height, prostate cancer, aggressiveness

## Abstract

**Background:**

The relationship between prostate cancer and height is uncertain.

**Methods:**

We prospectively examined the association of height with prostate cancer among 34268 men in the prostate, lung, colorectal, and ovarian cancer trial. Anthropometry was assessed at baseline and 2144 incident prostate cancer cases were identified upto 8.9 years of follow-up.

**Results:**

Overall, tallness was not associated with the risk of prostate cancer or with the risk of non-aggressive disease, but the risk for aggressive prostate cancer tended to be greater in taller men (Gleason score ⩾7 or stage ⩾III; *P* trend=0.05; relative risk (RR) for 190 cm+ *vs* ⩽170 cm=1.39, 95% confidence interval (95% CI): 0.96–2.01). This association was largely limited to men below the age of 65 years (*P* trend=0.008; RR for 190 cm+ *vs* ⩽170 cm=1.76, 95% CI: 1.06–2.93; *P* for interaction=0.009), although the number of cases was small and risk estimates were somewhat unstable.

**Conclusion:**

The results of this large prospective prostate cancer screening trial suggest that tallness is associated with increased risk for younger onset aggressive prostate cancer.

Tallness is a potential risk factor for prostate cancer (Macinnis and English, 2006), possibly because of increased levels of bioavailable insulin-like growth factors (IGFs) or androgens (Tanner, 1990), or to genetic predisposition (Silventoinen *et al*, 2003). This association is supported by several, but not all, population-based studies, with a modest pooled effect size (relative risk (RR)=1.09, 95% confidence interval (95% CI)=1.06–1.12, per 10-cm increase) based on 56 studies up to 2008 (Zuccolo *et al*, 2008). Since then, a large European cohort study (1500 cases; 129 502 men) reported a null association (RR=1.01, 95% CI=0.98–1.04 per 5-cm increase) (Pischon *et al*, 2008). It is unclear whether the association between height and prostate cancer differs according to tumour characteristics or other risk factors. In the Prostate, Lung, Colorectal, and Ovarian (PLCO) Cancer Screening Trial, which has a standardised prostate cancer screening protocol, we examined the relationship of height with total prostate cancer, non-aggressive prostate cancer, and with aggressive prostate cancer.

## Materials and methods

The PLCO Screening Trial is a multi-centre trial designed to evaluate screening methods for the early detection of these cancers (Prorok *et al*, 2000). Briefly, more than 75 000 men, aged 55–74 years, were recruited from 10 centres between 1993 and 2001 and randomised to receive either annual prostate screening (serum prostate-specific antigen (PSA) testing and digital rectal examination (DRE)) or standard care. Participants completed a risk factor questionnaire at baseline, which included current weight and height. The study was approved by the institutional review boards at the National Cancer Institute and the screening centres, and all participants provided informed consent.

Of the 38 349 men who were randomly assigned to the screening arm of the trial, we excluded men who reported a history of cancer other than non-melanoma skin cancer (*n*=775); men without an initial PSA test or DRE (*n*=2470); men who received an initial screening examination, but with whom there was no subsequent contact (*n*=721); men who did not complete the baseline questionnaire (*n*=898); and men with missing (*n*=205) or extreme values for height (*n*=12; <150 cm). We also excluded men whose initial screening examination occurred after 30 September 2002, the censoring date for this analysis (*n*=72). After exclusions, the analytic cohort comprised 34 268 men (some participants were included in more than one exclusion category).

For men with suspected prostate cancer from cancer screening or for those who reported prostate cancer on their annual follow-up questionnaire, we requested medical records to confirm the diagnosis and to obtain TNM stage and grade information. We used death certificates, autopsy reports, and supporting medical/pathological records to confirm diagnosis, stage, and grade for the deceased participants. Only histologically confirmed cases were included in the analysis. Prostate cancers were defined as non-aggressive if classified as stage I or II (localised disease) and had a Gleason sum of <7 (low-grade disease). Tumours were considered aggressive if classified as stage III or IV (high-stage disease) or assigned a Gleason sum of ⩾7 (high grade-disease). We also distinguished between high-grade disease (defined as a Gleason sum of ⩾7) and high-stage disease (defined as stage III or IV).

Person-years were calculated from the date of the baseline prostate cancer screening to the date of the most recently completed annual follow-up questionnaire, date of diagnosis, death, or 30 September 2002, whichever came first. We used Cox proportional hazards regression analysis to estimate RRs and 95% CIs. Multivariable analyses were adjusted for confounding factors, including age (continuous), race (White, Black, Asian/Pacific Islander, other), PLCO study centre, family history of prostate cancer (first degree; yes, no, missing), smoking status (never, current, former, pipe–cigar only), education (<12 years, 12 years of high school, post high school or college, and college graduation or more), history of diabetes (yes, no, missing), body mass index (BMI) (18.5–24.9, 25.0–29.9, 30.0 kg/ m^2^ or more), and number of screenings using PSA or DRE during the trial. Tests for linear trend were conducted by assigning the median value for each category and treating that term as a single continuous variable in the model. In addition, stratified analyses were carried out on the basis of age at baseline, history of prostate cancer, race, history of diabetes, current BMI, and BMI at age 18 years. We formally tested for interactions using log-likelihood ratio tests. All the analyses were conducted using SAS version 9.1 (SAS Institute Inc., Cary, NC, USA). All *P*-values were two sided.

## Results

Taller men were more likely to be Caucasian or African American, to have a positive family history of prostate cancer, to smoke, and to consume more total energy than shorter men (Table 1). Other characteristics, including the frequency of prostate cancer screenings during the trial, did not vary appreciably by height.

During 170 882 person-years of follow-up of 34 268 men, 2144 men were diagnosed with prostate cancer, of whom 1202 (57%) were classified as non-aggressive and 912 (43%) as aggressive cases. In multivariable analysis, height was associated neither with the risk of prostate cancer overall nor with non-aggressive disease (Table 2). However, the risk for aggressive prostate cancer tended to be greater in taller men, with risk increasing in a dose-response manner (Gleason score ⩾7 or stages ⩾III; *P* trend=0.05; RR=1.39, 95% CI=0.96–2.01; comparing 190+ with ⩽170 cm). Positive trends with height were noted for both high-grade (Gleason score ⩾7 only; *P* trend=0.04) and high-stage disease (stages ⩾III only; *P* trend=0.06).

As exploratory analyses, we have examined whether relationships with height differ according to prostate cancer risk factors. A strong positive association with aggressive disease was observed for men younger than 65 years (*P* trend=0.008; RR=1.76, 95% CI=1.06–2.93; comparing 190+ with ⩽170 cm). No clear pattern was observed for men in the older (⩾65 years) age group. The test for interaction between age and height was statistically significant (*P* interaction=0.009). There was no interaction between height and other risk factors (i.e., race, diabetes, current BMI, or BMI at age 18 years) in relation to risk (all *P* interactions >0.05; data not shown).

## Discussion

In this large prospective study, we found that height was associated with increased risk of aggressive prostate cancer in individuals below the age of 65 years. As all participants in this screening trial were screened annually using both PSA testing and DRE for the first 5 years on study, our findings are not likely to be due to differential cancer detection according to height. As all prostate cancers were evaluated uniformly for stage and grade by biopsy, the possibility of misclassification of cases was also minimal.

More than 30 prospective and 27 case–control studies have examined height and overall prostate cancer risk (Macinnis and English, 2006; Zuccolo *et al*, 2008), but findings are mixed; some studies showed a positive association (Le Marchand *et al*, 1994; Andersson *et al*, 1997; Giovannucci *et al*, 1997; Hebert *et al*, 1997; Rodriguez *et al*, 2001; Engeland *et al*, 2003; Cox *et al*, 2006; Sequoia *et al*, 2006; Zuccolo *et al*, 2008), whereas others found no relation (Severson *et al*, 1988; Cerhan *et al*, 1997; Veierod *et al*, 1997; Nilsen and Vatten, 1999; Clarke and Whittemore, 2000; Pischon *et al*, 2008). Stronger associations were observed for more advanced or fatal prostate cancer among 7 (Andersson *et al*, 1997; Giovannucci *et al*, 1997, 2004; Rodriguez *et al*, 2001; Cox *et al*, 2006; Sequoia *et al*, 2006; Zuccolo *et al*, 2008) of the 14 (Le Marchand *et al*, 1994; Andersson *et al*, 1997; Giovannucci *et al*, 1997, 2004; Nilsen and Vatten, 1999; Habel *et al*, 2000; Hsing *et al*, 2000; Norrish *et al*, 2000; Rodriguez *et al*, 2001; Macinnis *et al*, 2003; Cox *et al*, 2006; Sequoia *et al*, 2006; Pischon *et al*, 2008; Zuccolo *et al*, 2008) studies that included analyses for advanced or fatal prostate cancers. Consistent with our findings, a recent meta-analysis reported that the pooled estimate for aggressive or fatal prostate cancer was slightly higher than for all prostate cancers (RR for advanced or fatal prostate cancer=1.12, 95% CI=1.05–1.19, per 10-cm increase in height) (Macinnis and English, 2006; Zuccolo *et al*, 2008); however, the meta-analysis showed marked between-study heterogeneity (*I*^2^=47%) (Zuccolo *et al*, 2008).

Our finding of a stronger association with younger onset aggressive prostate cancer is consistent with a prospective study in Hawaii (<72.5 years, *n*=31 cases) (Le Marchand *et al*, 1994), but numbers were small and risk estimates unstable. No other study has reported on the height–prostate cancer relationship according to age. Two studies reported somewhat stronger associations with advanced/aggressive disease in men with a positive family history of prostate cancer (Norrish *et al*, 2000; Ahn *et al*, 2008). The limited numbers of aggressive cases with a first-degree family history (*n*=109), prevented our examining potential interaction with family history. Our findings require confirmation in large consortium-based studies.

Height is influenced by bioavailable IGFs and androgens (Tanner, 1990). Insulin-like growth factors (Roddam *et al*, 2008) and androgens (Henderson *et al*, 2003) have been implicated in prostate cancer, thus providing a biologically plausible link with height. However, the reasons for stronger associations with height in aggressive disease among younger than older men are not clear. Further biochemical studies investigating the underlying potential biological mechanisms are warranted.

A limitation of our study is that height was assessed by self-report and hence was not validated. Nevertheless, the correlations between measured and self-reported height are typically high, ranging from 0.80 to 0.95 (Willett, 1998), although men tend to overreport their height (Spencer *et al*, 2002); which could have attenuated estimates of effect toward the null. Advantages of this study include its large size, prospective design, and reasonably detailed anthropometry data with a substantial range in height levels. This enabled us to examine the risk of prostate cancer according to narrow height categories across numerous potentially important effect modifiers.

This large prospective study suggests that tallness may be associated with increased risk of aggressive prostate cancer, particularly in younger men; confirmation is needed in other populations.

## Figures and Tables

**Table 1:** Baseline characteristics of participants according to height, PLCO study^a^

	**Height (cm)**					
**Characteristic**	**⩽170**	**171–174.9**	**175–179.9**	**180–184.9**	**185–189.9**	**190+**
Number of participants, *n* (%)	3472 (10.1)	6472 (18.9)	9804 (28.6)	8773 (25.6)	4276 (12.5)	1471 (4.3)
Age, years	63.4	63.1	62.7	62.3	61.9	61.7
						
*Race, %*						
White	66.3	85.0	91.9	93.3	94.0	91.7
Black	4.8	4.0	4.0	4.4	4.7	6.5
Others	28.9	11	4.1	2.3	1.3	1.8
						
Family history of prostate cancer, %	7.2	7.6	7.2	7.5	8.3	8.7
History of diabetes, %	9.9	10.5	8.4	9.1	8.0	10.3
Daily aspirin use, %	29.3	30.9	30.5	30.7	29.6	28.9
						
*Smoking status, %*						
Never	32.2	29.5	29.1	29.0	28.3	28.2
Current	10.2	11.2	11.0	11.4	11.7	10.9
Former	50.1	51.4	52.1	51.8	51.9	52.3
Cigar or pipe only	7.5	7.9	7.9	7.8	8.2	8.6
						
Physical activity, h per week	1.9	2.0	2.0	2.0	2.0	2.0
Total energy, kcal per day	2184	2280	2352	2412	2503	2589
Current BMI, kg m^−2^	27.4	27.5	27.6	27.6	27.6	27.5
BMI at age 20, kg m^−2^	23.0	22.9	23.0	23.0	22.9	22.6
						
PSA (baseline), ng ml^−1^	2.4	1.8	1.8	1.9	2.2	1.7
DRE (baseline), %	6.7	7.6	8.1	7.4	7.3	6.1
Biopsy (before study participation), %	4.5	4.0	4.3	4.4	4.0	3.9
No. of screens per year^b^	0.8	0.8	0.8	0.8	0.8	0.8

BMI=body mass index; DRE=digital rectal examination; PLCO=Prostate, Lung, Colorectal, and Ovarian; PSA=prostate-specific antigen.

a Means or proportions.

b Average number of prostate cancer screening examinations (PSA test and/or DRE) during the period of active screening (years: 0–5).

**Table 2:** Multivariate relative risks (RR) and 95% confidence intervals (CIs) of prostate cancer in relation to height, PLCO study

**Height (cm)**	**⩽170**	**171–174.9**	**175–179.9**	**180–184.9**	**185–189.9**	**190+**	**5 cm increment**	***P* trend**
*Total study population*								
No. total cases	202	387	619	570	275	91		
RR (95% CI)^a^	1.00 (ref)	1.06 (0.89–1.26)	1.07 (0.91–1.26)	1.14 (0.96–1.34)	1.10 (0.91–1.33)	1.06 (0.82–1.36)	1.02 (0.98–1.05)	0.28
No. non-aggressive cases^b^	110	212	358	331	148	43		
RR (95% CI)^a^	1.00 (ref)	1.04 (0.82–1.31)	1.09 (0.87–1.35)	1.16 (0.93–1.46)	1.04 (0.81–1.35)	0.88 (0.61–1.26)	1.00 (0.96–1.05)	0.92
No. aggressive cases^b^	84	170	255	232	125	46		
RR (95% CI)^a^	1.00 (ref)	1.17 (0.90–1.53)	1.14 (0.88–1.48)	1.19 (0.91–1.54)	1.29 (0.97–1.73)	1.39 (0.96–2.01)	1.05 (1.00–1.10)	0.05
								
*Age <65 years*								
No. aggressive cases^b^	32	69	119	128	68	30		
RR (95% CI)^a^	1.00 (ref)	1.17 (0.76–1.78)	1.23 (0.82–1.84)	1.38 (0.93–2.06)	1.47 (0.95–2.26)	1.76 (1.06–2.93)	1.08 (1.01–1.15)	0.008
								
*Age ⩾65 y*ears								
No. aggressive cases^b^	52	101	136	104	56	16		
RR (95% CI)^a^	1.00 (ref)	1.20 (0.85–1.70)	1.08 (0.77–1.52)	1.02 (0.72–1.46)	1.16 (0.78–1.72)	1.09 (0.62–1.95)	1.01 (0.95–1.09)	0.88

a Relative risk adjusted for age, race, family history of prostate cancer, study centre, education, smoking, diabetes, total energy intake, and current body mass index, and number of screenings using prostate specific antigen or digital rectal examination during the trial.

b Non-aggressive cases were defined as a Gleason score of <7 and stage I/II. Aggressive cases were defined as a Gleason score of 7+ or stage III+. The number of non-aggressive cases plus the number of aggressive cases does not equal the number of total cases because 30 cases were deleted due to missing data on Gleason sum or tumour stage.
